# Innovations in Liver Preservation Techniques for Transplants from Donors after Circulatory Death: A Special Focus on Transplant Oncology

**DOI:** 10.3390/jcm13185371

**Published:** 2024-09-11

**Authors:** Michele Finotti, Maurizio Romano, Ugo Grossi, Enrico Dalla Bona, Patrizia Pelizzo, Marco Piccino, Michele Scopelliti, Paolo Zanatta, Giacomo Zanus

**Affiliations:** 1 Hepatobiliary and General Surgery Unit, Regional Hospital Treviso, Dipartimento di Scienze Chirurgiche Oncologiche e Gastroenterologiche (DISCOG), University of Padua, 35128 Padua, Italy; maurizio.romano@aulss2.veneto.it (M.R.); ugo.grossi@aulss2.veneto.it (U.G.); patrizia.pelizzo@aulss2.veneto.it (P.P.); marco.piccino@aulss2.veneto.it (M.P.); michele.scopelliti@aulss2.veneto.it (M.S.); giacomo.zanus@aulss2.veneto.it (G.Z.); 2 Simmons Transplant Institute, Baylor University Medical Center, Dallas, TX 75246, USA; 3 Department of Anesthesiology and Critical Care, Treviso Regional Hospital AULSS 2 Marca Trevigiana, 31100 Treviso, Italy; paolo.zanatta1@aulss2.veneto.it

**Keywords:** machine perfusion, marginal graft, transplant oncology, cancer recurrence

## Abstract

Liver transplantation is the preferred treatment for end-stage liver disease. Emerging evidence suggests a potential role for liver transplantation in treating liver tumors such as colorectal liver metastases and cholangiocarcinoma. However, due to a limited donor pool, the use of marginal grafts from donation after circulatory death (DCD) donors is increasing to meet demand. Machine perfusion is crucial in this context for improving graft acceptance rates and reducing ischemia–reperfusion injury. Few studies have evaluated the role of machine perfusion in the context of transplant oncology. Perfusion machines can be utilized in situ (normothermic regional perfusion—NRP) or ex situ (hypothermic and normothermic machine perfusion), either in combination or as a complement to conventional in situ cold flush and static cold storage. The objective of this analysis is to provide an up-to-date overview of perfusion machines and their function in donation after circulatory death with particular attention to their current and likely potential effects on transplant oncology. A literature review comparing standard cold storage to machine perfusion methods showed that, so far, there is no evidence that these devices can reduce the tumor recurrence rate. However, some evidence suggests that these innovative perfusion techniques can improve graft function, reduce ischemia–reperfusion injury, and, based on this mechanism, may lead to future improvements in cancer recurrence.

## 1. Introduction

Donors after circulatory death (DCD) utilization decreased after donation after brainstem death (DBD) became legal, due to the superior quality of DBD organs. However, some US institutions began to revive DCD organ transplantation in the 1990s to reduce the chronic gap between supply and demand in transplantation.

As the need for donors increased and DCD donation became more ethically and medically acceptable, the number of DCD donors grew from 41 in 1993 to 4190 in 2021 [1,2].

Even though the use of organs from DCD donors has been steadily increasing in the US, there is still room for expansion to bridge the gap between supply and demand for transplantation. DCD livers have always been considered a marginal graft with high postoperative risks, particularly associated with ischemic cholangiopathy and the potential need for re-transplantation [3]. In this context, the utilization of in situ/ex situ machine perfusion can significantly enhance DCD quality and utilization, particularly for non-common indications such as transplant oncology [4]. This narrative review assesses the role of DCD donation in liver transplant, with emphasis on the use of machine perfusion devices. This article examines the advancements in liver preservation techniques and their influence on transplant oncology, which includes both primary liver cancers (such as hepatocellular carcinoma (HCC)) and secondary liver tumors, including colorectal liver metastases (CRLM), in animal and human models. The discussion extends to the potential impact of these advancements, particularly machine perfusion, on tumor recurrence and graft survival, depending on the tumor stage.

## 2. The Role of New Technologies in DCD Donation

The standard protocol for organ recovery from DCD donors involves discontinuing life-sustaining measures, including mechanical ventilation, resulting in circulatory arrest (agonal phase). A variable observation period is required before determining the donor’s death. A prompt sternotomy and laparotomy enable rapid vessel cannulation and in situ perfusion of the organs with cold preservation solutions (super-rapid recovery, SRR). Organs experience the so-called donor warm ischemia time (DWIT), which causes ischemic injury linked to harmful transplant results based on the length of the DWIT. For example, in liver transplantation, long DWIT may lead to ischemic cholangiopathy while in kidney transplantation and then to delayed graft function, both of which pose an increased risk of organ discard [5,6]. Additionally, the use of the rapid recovery technique may increase the risk of organ injury [7,8].

In this context, various techniques have been suggested to enhance graft results and minimize organ ischemia in DCD donation, such as perfusion devices.

Perfusion machines can be used in situ, such as normothermic regional perfusion (NRP), or ex situ such as hypothermic and normothermic machine perfusion, in combination, as an alternative, or as a complement to conventional in situ cold flush and static cold storage [9].

The goal is to reduce graft injury, reduce exposure to hypoxia, improve quality and duration of preservation, and allow for potential graft assessment to increase organ utilization. These methodologies have predominantly been assessed in Europe and are increasingly being implemented in the US [10]. These technologies require high investment with an important impact on healthcare finances, as pointed out in a recent paper by Carvalho et al. in *Hepatology*. The study highlights two important needs to start a program with machine perfusion devices: financial and clinical resources. In the United States, the cost of a single perfusion procedure can reach $100,000, particularly when all necessary services are included. Future cost-analysis studies with long-term evaluations are essential to identify in which types of patients and conditions these perfusion machines are most beneficial. While hypothermic devices are less expensive, they remain costly. We must consider not only the financial aspects but also the elevated use of human resources required by machine perfusions. Liver perfusion is complex, particularly when initiated at the donor site, requiring significant surgical expertise. Although modern devices are designed to be user-friendly, they remain complicated, making adequate staff training essential [11].

Each form of machine perfusion offers advantages and disadvantages, as outlined in the ensuing sections.

### 2.1. Normothermic Regional Perfusion

Normothermic regional perfusion (NRP) is a post-mortem organ perfusion technique that utilizes oxygenated blood to recondition abdominal and/or thoracic organs before cold preservation. NRP exclusively perfuses a specific region (abdomen [A-NRP] or chest and abdomen [TA-NRP]) while excluding upper and lower extremities and cerebral circulation through clamps or occlusion balloons. The venous system of the donor is accessed, drained, and then passed through a membrane oxygenator before being pumped back into the arterial circulation, facilitated by the use of extracorporeal membrane oxygenation (ECMO) devices, which help to maintain organ viability [11,12].

The benefits of NRP have been demonstrated in various centers across different organs. Messer and colleagues reported a study on heart transplantation that compared 75 donors who underwent rapid DCD recovery with 25 who underwent TA-NRP. The results showed that TA-NRP was associated with significantly better graft and patient survival [13]. Additionally, NRP DCD heart recipients necessitated decreased ventilatory support, experienced decreased rates of postoperative hemodialysis, and demonstrated a shorter median length of stay compared to SRR DCD heart recipients. Hoffman et al. presented a collection of heart transplants that used TA-NRP followed by static cold storage (SCS), resulting in significant cost savings over perfused hearts and facilitating interhospital transport logistics [14]. From a perspective centered on cardiac DCD, NRP permits functional in situ evaluation of the heart, can be used in conjunction with machine perfusion or SCS, and has the potential to expand transplant center acceptance of a wider range of donors (e.g., hearts from DCD donors > 50 years of age).

NRP also benefits the organs in the abdominal area [15,16]. Compared to SRR DCD, liver transplantation using NRP DCD is linked to reduced early allograft dysfunction (EAD) rates (12% for NRP versus 32% for non-NRP livers), 30-day graft loss (2% NRP livers versus 12% non-NRP livers), fewer anastomotic strictures (7% versus 27% for non-NRP, *p* = 0.0041), and decreased IC rates (0% versus 27%, *p* < 0.001). As with DCD heart donors, NRP-DCD allows for improved evaluation of liver grafts that may have been discarded based on pre-retrieval information or post-cross clamp assessment after rapid recovery technique. Various parameters, including serum lactate, liver transaminases, glucose metabolism, and pH, can be assessed during the NRP to evaluate the quality of the liver graft [11,17,18,19,20]. The tremendously promising outcomes of NRP for liver transplantation, which exhibit comparable extended-term overall graft survival in certain series of DBD donations, are advocating for transplant centers to reconsider labeling DCD donors as “extended criteria” [21]. For the aforementioned reasons, centers in the UK and France regularly utilize NRP for multiorgan DCD procurements. Additionally, NRP is obligatory for DCD organ procurement in France, and the use of NRP in DCD donors is steadily increasing in other European countries, particularly in Spain and Italy, where it is often combined with ex vivo machine perfusion techniques to further enhance organ preservation [22,23].

### 2.2. Ex Situ Machine Perfusion

Ex situ machine perfusion (MP) provides advantages over static cold storage (SCS) in assessing and improving the quality of liver grafts. MP offers oxygen and nutrients, leading to better organ function and reducing the risk of ischemia–reperfusion injury, postoperative graft failure, early allograft dysfunction, and biliary complications. Moreover, MP techniques allow for pre-implantation evaluations of graft function and quality [24,25].

### 2.3. Hypothermic MP

In liver transplantation, Guarrera et al. initially described using ex situ hypothermic machine perfusion (H-MP) without active oxygenation at a temperature of 4–6 °C through the portal vein and hepatic artery. They were able to successfully perform clinical transplantation with an extended criteria donation after brain death (DBD) donor [26].

Dutkowski and colleagues conducted a study on hypothermic (10 °C) oxygenated perfusion via the portal vein of DCD livers. Results indicated a significant decrease in graft injury when compared to matched SCS DCD livers, as well as lower peak alanine aminotransferase, a lower intrahepatic cholangiopathy rate (0% vs. 22%, *p* = 0.015), and better 1-year graft survival (90% vs. 69%, *p* = 0.035). The H-MP cohort showed zero re-transplantations, while 18% of SCS DCD liver recipients required a re-transplantation in this study [27]. Schlegel and colleagues conducted a 5-year follow-up study on 50 livers from DCD. The livers were divided into two groups: those with 1–2 h of hypothermic machine perfusion (H-MP) after SCS and those with only SCS. Despite a higher UK DCD risk score in the H-MP group due to longer DWIT and older donors, the recipients showed statistically significant lower acute rejection rates, less primary non-function, less ischemic cholangiopathy, and higher 5-year graft survival (94% H-MP-DCD, 78% SCS-DCD, *p* = 0.024) [28].

Van Rijn and colleagues utilized active oxygenation perfusion through both the portal vein and the hepatic artery in their study on dual hypothermic oxygenated machine perfusion (D-HOPE), resulting in significant improvements in restoring hepatic adenosine triphosphate, reducing reperfusion injury, and increasing graft survival rates [29]. The authors conducted a randomized multicenter study involving 160 DCD livers to investigate the effect of D-HOPE versus SCS. The results show that D-HOPE resulted in a significantly lower non-anastomotic biliary stricture rate (6% vs. 18%), EAD rate (26% vs. 40%), and post-reperfusion syndrome rate (12% vs. 27%). The two groups exhibited comparable rates of patient and graft survival and graft utilization, but the cumulative number of interventions or antibiotic therapy for non-anastomotic biliary strictures was quadruple higher in the SCS group as opposed to the D-HOPE group [30]. Two significant limitations of hypothermic MP are that it does not recreate physiologic reperfusion of the liver graft and does not allow real-time assessment of graft viability.

### 2.4. Normothermic MP

Normothermic machine perfusion (NMP) aims to prevent preservation injury resulting from cooling and hypoxia associated with both H-MP and SCS [31]. NMP operates at a temperature of 37 °C; provides nutrients, and oxygen; restores adenosine triphosphate levels; reduces reactive oxygen species accumulation; and facilitates graft metabolic activity. The first clinical randomized controlled trial comparing NMP to SCS in DBD and DCD liver transplantation was published by Nasralla et al. Seven centers participated, with a total of 220 transplanted livers, 53 of which were DCD. The analysis of a subgroup of DCD livers perfused with NMP, as compared to SCS, revealed a 49% reduction in peak aspartate transaminase levels (*p* < 0.0001) and a 74% lower risk of EAD among NMP recipients (*p* = 0.001). Moreover, NMP was linked to a 50% increase in the utilization of DCD grafts (*p* = 0.01) and a 54% increase in preservation time (*p* < 0.0001) [32].

NMP not only enhances quality but also enables evaluating graft viability during perfusion via numerous indicators comprising pH, lactate, bile composition, perfusate aspartate transaminase/alanine aminotransferase ratio, and flow. In their study, Mergnetal et al. demonstrated that NMP’s capacity to assess graft quality boosted DCD graft utilization [19]. Thirty-one high-risk livers, including 14 from DCD, were declined by all transplant centers in the United Kingdom. These livers were then perfused with normothermic machine perfusion (NMP) for four hours, during which the pH, lactate, glucose, bile, and vascular flows were evaluated. Of these, 22 livers (71%) were successfully transplanted after a median of 18 h of preservation, and all patients survived at three months post-transplantation. However, 18% of the recipients developed ischemic cholangiopathy (IC) and 30% required re-transplantation [19]. However, it is possible that the high rates of biliary ischemic injury can be attributed to the median of over 8 h of SCS that the grafts underwent before NMP.

A recent multicenter randomized clinical trial compared the outcomes of 300 recipients of DBD and DCD livers preserved using either a portable NMP (OCS liver console) or SCS. The portable system, known as back-to-base NMP, initiates perfusion at the donor hospital and eliminates SCS. Results showed that NMP-DCD livers had a significantly greater utilization rate of 51% compared to 26% with fewer ischemic biliary complications at 12 months (2.6% vs. 9.9%, *p* = 0.02). This study suggests that initiating NMP early and avoiding SCS could potentially reduce biliary ischemic injury in DCD liver grafts [33].

### 2.5. Combining Perfusion Strategies

NRP, NMP, and H-MP can enhance the utilization of DCD liver grafts and improve recipient outcomes individually. However, it is still unclear how and if a combination of these technologies can produce better results, as well as which combinations would be most beneficial. Early animal studies suggested the feasibility of combining in situ and ex situ techniques. Fondevilla et al. explored the sequential usage of in situ NRP and ex situ NMP in a large animal model, delivering remarkable results even after prolonged warm ischemia. The donor pigs experienced cardiac arrest for 90 min and were split into three groups. In the first group, livers were preserved right away with SCS. In the other two groups, donors underwent a 60 min NRP followed by either SCS (NRP + SCS) or MP (NRP + MP). After 4 h of preservation, the livers were transplanted into recipient pigs. The survival rate after five days was 0% in SCS, 83% in NRP + SCS, and 100% in NRP + MP [34].

Ghinolfi and colleagues validated these findings in a clinical trial on human liver transplantation conducted in Italy. In Italy, for DCD cases, a continuous electrocardiogram flat-line of twenty minutes is required for a declaration of death, leading to a prolonged warm ischemia time. During the study, 34 liver DCD donors were considered. Of these, three were discarded before NRP, and 11 were excluded based on NRP parameters or the liver’s macroscopic appearance at procurement, biopsy results, or evidence of severe macroangiopathy at back table evaluation. A total of 20 liver grafts underwent ex situ perfusion, with 9 using normothermic machine perfusion and 11 using dual hypothermic machine perfusion. The median warm ischemia time of the grafts was 52.5 (range: 47–74) minutes. The study transplanted 18 livers without primary non-function (PNF) and 5 cases of early allograft dysfunction (EAD). After a median follow-up of 15.1 months (range: 9.5–22.3 months), one case of ischemic-type biliary lesion and one patient death were reported. The study demonstrated the effectiveness of NRP + MP in enhancing the preservation and recovery of donation after DCD liver grafts, even with a prolonged warm ischemia time [35]. In 2020, Muller et al. reported the first large-scale international multicenter comparison of outcomes after NRP and HOPE in DCD liver transplantation, which revealed comparable 1-year graft survival rates (93% vs. 87%). Additionally, there was no variation in cholangiopathy, but greater organ utilization was observed in the HOPE group (81% vs. 63%) [36].

Because temperature is a continuous variable, the study by Hoyer and colleagues investigated a controlled oxygenated rewarming strategy (COR) following cold storage. Eighteen patients underwent machine-assisted slow COR for 120 min before transplantation, demonstrating a 5-year patient survival rate of 93.8% compared to 75.8% in the control group [37].

A prospective clinical trial evaluated 16 DCD declined livers treated with ex situ NMP (viability assessment phase), preceded by 1 h D-HOPE (resuscitation phase) and 1 h COR, using a perfusion fluid containing a hemoglobin-based oxygen carrier. The NMP phase evaluated liver function, including perfusate lactate, pH, bile production, and bile pH. As per the results, 11 livers were successfully transplanted, with 100% patient and graft survival at 3 and 6 months [38].

## 3. The Role of New Technologies in Transplant Oncology in DCD Donation

Due to the current shortage of donor organs, extended criteria donors (ECDs), such as donation after circulatory death (DCD) donors, are becoming increasingly important in the field of transplant oncology. This is particularly true for patients with hepatocellular carcinoma (HCC) [39] and a low MELD score [40,41]. ECD will likely become the primary source of liver grafts for patients who are eligible for liver transplant, especially those with unresectable colorectal liver metastases (CRLM), intrahepatic cholangiocarcinoma, and Klatskin tumors [42]. It is widely recognized that ECD have lower tolerance to ischemia/reperfusion injury (IRI), as demonstrated in multiple studies [43,44]. Additionally, various animal and human studies have suggested a causal relationship between IRI and tumor recurrence, resulting from endothelial damage and increased growth signals (Table 1). A pair of studies by Oldani et al. have focused on the effect of ischemic liver transplants on the potential for cancer recurrence. These animal studies replicate in vivo and ex vivo perfusion, resulting in decreased IR injury and HCC recurrence compared to non-perfused livers. Studies indicate that liver steatosis, another feature that contributes to marginal grafts, exacerbates IR injury and increases the risk of severe HCC recurrence, as demonstrated by Orci et al. [45].

**Table 1:** The relationship between ischemic reperfusion injury (IRI) and tumor recurrence in animal and human models.

Authors	Type of Cancer	Model	Cell Type	Intervention	Duration	Conclusion
Doi et al. [46]	colorectal cancer	rat model	rat colon adenocarcinoma cells (RCN-H4)	30 or 60 min of 70% partial hepatic ischemia	3 weeks	I/R promoted liver metastasis and induced the expression of E-selectin mRNA
Yoshida et al. [47]	colorectal cancer	rat model	rat colon adenocarcinoma cells (RCN-H4)	group A (control), received laparotomy for 120 min with no liver ischemia; group B (continuous I/R), received 60 min of 70% partial liver ischemia followed by 60 min of reperfusion; and group C (intermittent I/R), which received 15 min of 70% ischemia and 15 min of reperfusion, repeated four times	3 weeks	continuous I/R (B) greatly promoted liver metastasis in both ischemic and nonischemic liver lobes, whereas intermittent I/R (C) showed significantly fewer metastasis than group B in both lobes. Significantly less E-selectin mRNA was expressed in group C than in group B
Ling et al. [48]	HCC	human model	/	37 patients with a ratio of GW > 60% (large graft group) and 78 patients received a graft GW < 60% (small graft group.)	/	patients with small-for-size liver grafts (<60% of standard liver weight, SLW) had significantly higher HCC recurrence (*p* = 0.04)
Oldani et al. [49]	HCC	rat model	HCC cells	control standard donors vs. 10 min or 30 min inflow liver clamping before retrieval vs. 2 h liver reperfusion after the clamping	2 weeks	HCC growth was higher in the 10 min and 30 min clamping and was prevented after 2 h reperfusion
Hamaguchi et al. [50]	HCC	rat model	N1S1 tumor cell implantation	different duration of hepatic pedicle clamping after major hepatectomy	3 weeks	longer clamping followed by reperfusion accelerated hepatocellular carcinoma growth
Orci et al. [51]	HCC	rat model	Hepa 1–6 HCC cells	portal and arterial vascular liver inflow clamping	3 weeks	increase in hepatocellular damage and expression of inflammatory genes, >in mice with severe steatosis
Wang et al. [52]	HCC	rat model	CBRH-7919 cells	inflow liver clamping and inhibition of factors increasing IR injury (PARP-1)	/	IR-induced PARP-1 up-regulation increasing HCC recurrence
Orci et al. [53]	HCC	rat model	RIL-175 cells	occlusion of the hepatic and femoral blood vessels	3 weeks	portal triad clamping provoked increased bacterial translocation, resulting in aggravated tumor burden
Oldani et al. [45]	HCC	rat model	JM-1 cells	DCD grafts were perfused for 2 h with hypothermic or normothermic perfusion and transplanted vs. non-perfused liver	4 weeks	non-ex vivo perfused DCD liver recipients constantly developed larger tumors
Yang et al. [54]	HCC	rat model		inflow liver clamping	3 weeks	higher HCC recurrence in NAFLD
van der Bilt et al. [55]	CRLM	rat model	C26 colon carcinoma cells	inflow liver clamping	12 days	the outgrowth of micrometastases in occluded liver lobes was accelerated five- to six-fold compared with nonoccluded lobes
van der Bilt et al. [56]	CRLM	rat model	C26 colon carcinoma cells	inflow liver clamping for 45 min	5 days	I/R-stimulated tumor growth by more than 70%
Ogawa et al. [57]	HCC	rat model	McA-RH7777 cells	liver transplant with I/R and tacrolimus exposure	/	I/R and tacrolimus enhance the invasiveness of HCC
Man et al. [58]	HCC	rat model	McA-RH7777 cells	partial hepatic I/R injury with or without major hepatectomy	4 weeks	significant tumor growth and intrahepatic metastasis were found in rats undergoing I/R and major hepatectomy
Nicoud et al. [59]	CRLM	rat model	MC38 cell	30 min of 70% liver ischemia	4 weeks	liver with ischemia at the time of tumor inoculation had significantly larger tumor number and volume
Man et al. [60]	HCC	rat model	McA-RH7777 cells	liver transplant with whole and small-size grafts	6 weeks	small-for-size liver grafts promote tumor growth and metastasis after liver transplantation
Man et al. [61]	HCC	rat model	McA-RH7777 cells	liver transplant with whole and small-size grafts	6 weeks	small-for-size liver grafts promote tumor growth and metastasis after liver transplantation associated with CXCL10 overexpression

CRLM: colorectal liver metastases; DCD: donors after circulatory death; HCC: hepatocellular carcinoma; I/R: ischemic reperfusion Injury.

If the clear correlation between IRI and HCC in LT is evident in experimental models, it remains a subject of debate due to inconsistent human data (refer to Table 2).

For instance, Silverstein and colleagues evaluated the impact of DCD use on hepatocellular carcinoma (HCC) recurrence via a retrospective study utilizing the UNOS database. The study compared 6996 liver transplants from DBD to 567 from DCD donors in patients with similar HCC characteristics, such as alpha-fetoprotein level, tumor size and number, and bridge/downstaging treatments. The study revealed that the rate of HCC recurrence was comparable in both populations after a median follow-up of 2.1 years post liver transplant [62]. Prior research has demonstrated comparable rates of patient and graft survival as well as HCC recurrence [63,64,65,66]. However, patient and graft survival was inferior in DCD donors compared to DBD donors when analyzing the high-risk HCC recurrence population (AFP at LT > 100 ng/mL, progressive disease after LRT, or a RETREAT score > 4) [62].

With the advent of in vivo and ex vivo machine perfusion, there is growing interest in their potential to reduce ischemia–reperfusion (IR) injury in marginal grafts and potentially lower the risk of tumor recurrence.

Rigo et al. conducted a single-center retrospective cohort study to compare the rate of HCC recurrence following liver transplantation with grafts stored in standard cold storage versus D-HOPE. The study included 246 and 80 patients transplanted with grafts preserved in SCS and D-HOPE, respectively. With a minimum follow-up period of two years and comparable HCC characteristics and downstaging/bridge therapy, the entire cohort had an HCC recurrence rate of 9.2%, with no significant difference between SCS and D-HOPE [44].

However, the role of D-HOPE in preventing HCC recurrence may be underestimated as it can compensate for the marginality of the graft. The graft in the D-HOPE group is more marginal than in SCS due to factors such as donors with older age, higher BMI, and higher degree of macrovesicular steatosis. These factors increase susceptibility to IRI and possibly higher HCC recurrence. Additionally, only 17% of the grafts considered in the study were from DCD donors [44].

In contrast, Muller and colleagues demonstrated that machine perfusion may lower the HCC recurrence rate. Specifically, the group examined 140 individuals who underwent HCC transplantation, 70 from DBD and 70 from DCD donors with HOPE-treatment. There were no differences in tumor burden or pre-LT treatment between HOPE-treated DCD and non-perfused DBD liver recipients, as well as in the operative LT technique, postoperative immune suppression, and donor characteristics. The research revealed a reduction in HCC recurrence among the HOPE DCD group compared to the non-perfused DBD donors, with rates of 5.7% and 25.7%, respectively (*p* = 0.002). Moreover, the HOPE group experienced higher rates of recipient survival free from recurrence, with 92% versus 73% after 5 years (*p* = 0.027). These findings are further supported by the improved function of the graft and lower systemic inflammatory response observed in the HOPE group [67]. As pointed out by recent meta-analyses, HOPE and NMP are associated with lower rates of early allograft dysfunction, with HOPE linked to lower re-transplantation rates and better graft survival. However, the follow-up period is limited to 1 year [68].

**Table 2:** Relationship between ischemic reperfusion injury (IRI) and tumor recurrence in human models.

Authors	Year	Type of Study	Machine Perfusion	Patients	HCC Stage	Median Follow up	Patient Survival	Graft Survival	HCC Recurrence
Silverstein et al. [62]	2020	Retrospective	No	6996 DBD vs. 567 DCD	Comparable	2.1 years	81.1% for DCD vs. 85.5% DBD recipients at 3 years (*p* = 0.008)	76.3% for DCD vs. 83.5% DBD recipients at 3 years (*p* < 0.001	7.6% for DCD vs. 6.4% DBD recipients at 3 years (*p* = 0.67)
Croome et al. [65]	2015	Retrospective	No	340 DBD vs. 57 DCD	Comparable	3.9 years	75% for DCD vs. 80% DBD recipients at 3 years (*p* = 0.07)	n.a.	12.3% for DCD and 12.1% for DBD (*p* = 0.91)
Goldkamp et al. [69]	2015	Retrospective	No	33 DBD vs. 11 DCD	Comparable	n.a.	Similar DCD vs. DBD	n.a.	0% for DCD and 9% for DBD (*p* = 0.4)
Martinez-Insfran et al. [70]	2019	Retrospective	No	18 DBD vs. 18 DCD	n.a.	17 months	DCD 75% vs. DBD 88% at 1 year	n.a.	n.a.
Khorsandi et al. [64]	2016	Retrospective	No	91 DBD vs. 91 DCD	no	1 year	Equivalent 1-, 3-, and 5-year OS (*p* = 0.115)	n.a.	Equivalent cancer-specific survival (*p* = 0.7)
Wallace et al. [63].	2022	Retrospective	No	830 DBD vs. 375 DCD	n.a.	n.a.	Equivalent	Equivalent	n.a.
Rigo et al. [44]	2023	Retrospective	D-HOPE	246 SCS-Vs 80 D-HOPE graft (14 DCD)	Comparable	2 years	n.a.	n.a.	9.2% with no difference SCS vs. D-HOPE
Mueller et al. [67]	2020	Retrospective	D-HOPE	70 non-perfused DBD vs. 70 DCD H-HOPE grafts	Comparable	5 years	n.a.	n.a.	5.7% vs. 25.7% (*p* = 0.002) HOPE DCD vs. non-perfused DBD

DBD: donation after brain death; DCD: donation after circulatory death; HCC: hepatocellular carcinoma; D-HOPE: dual hypothermic oxygenated perfusion; I/R: ischemic reperfusion injury; SCS: static cold storage.

## 4. Conclusions and Future Prospective

DCD donation is a vital resource for expanding transplantation opportunities across all organs. Innovative techniques, including NRP, H-MP, and NMP, have the potential to enhance graft evaluation and quality, thereby boosting graft utilization rates. DCD donors are a particularly valuable source of grafts, especially for “marginal” indications, such as in transplant oncology, and can serve as important tools in increasing the donor pool. It is important to note that the outcome measures are not homogeneous among studies. The outcomes studied can vary from postoperative complication rates and length of hospital stay to early graft dysfunction rates, re-transplant rates, and graft and overall survival, as well as the follow-up period, which is usually short.

In particular, the role of ischemic/reperfusion injury in tumor recurrence after liver transplantation is still unknown. As demonstrated in this review, further research is necessary to elucidate the function of in vivo/ex vivo machine perfusion in decreasing the potential risk of tumor recurrence.

## Data Availability

The data presented in this study are available on request from the corresponding author.
